# Ruptured ovarian cystic teratoma in pregnancy with diffuse peritoneal reaction mimicking advanced ovarian malignancy: a case report

**DOI:** 10.1186/1752-1947-2-203

**Published:** 2008-06-12

**Authors:** Sachchidananda Maiti, Zamurrad Fatima, ZK Anjum, RE Hopkins

**Affiliations:** 1 Department of Obstetrics and Gynaecology, St Mary's Hospital, Manchester M13 0JH, UK; 2 Department of Obstetrics and Gynaecology, Royal Bolton Hospital, Bolton, UK; 3 Department of Obstetrics and Gynaecology, Royal Oldham Hospital, Oldham OL1 2JH, UK; 4 Women Health Directorate, Royal Bolton Hospital, Bolton, UK

## Abstract

**Introduction:**

This case illustrates the unusual complication of granulomatous peritonitis following rupture of a dermoid cyst in pregnancy resembling disseminated ovarian carcinoma. To the best of the authors' knowledge, this is the first report of this complication during advanced pregnancy in the literature.

**Case presentation:**

A dermoid cyst ruptured during surgical removal in the second trimester of pregnancy in a 27-year-old primigravida. Postoperatively the patient suffered pulmonary embolism and leakage of sebaceous material through the abdominal wound. She gradually developed significant abdominal distension, gastrointestinal symptoms and lost more than 8 kg of weight in the 12 weeks postoperatively. The baby was delivered at 31 weeks by a technically challenging caesarean section owing to severe dense adhesions obscuring the uterus. Bowel resection was performed for suspected malignant infiltration and adhesion causing obstruction. She had a protracted convalescence with an ileostomy and mucus fistula. Histology confirmed granulation without malignancy. One year following the surgical treatment, she had recovered well and was planning her next pregnancy.

**Conclusion:**

Although granulomatous peritonitis following rupture of a dermoid cyst is very rare, awareness is the key to diagnosis and appropriate management. Per-operative frozen section may be helpful.

## Introduction

Mature cystic teratoma (dermoid cyst) is one of the most common benign ovarian neoplasms discovered during pregnancy (24–40%) [1,2]. They may be responsible for complications such as torsion, rupture and obstruction during labour. Rupture is rare, but once it has occurred it can cause complications such as chemical or granulomatous peritonitis mimicking advanced ovarian malignancy [3-5]. This case illustrates the unusual complication of granulomatous peritonitis following rupture of a dermoid cyst in pregnancy resembling disseminated ovarian carcinoma. To the best of the authors' knowledge, this is the first report of this complication during advanced pregnancy in the literature.

## Case presentation

**Figure 1:**
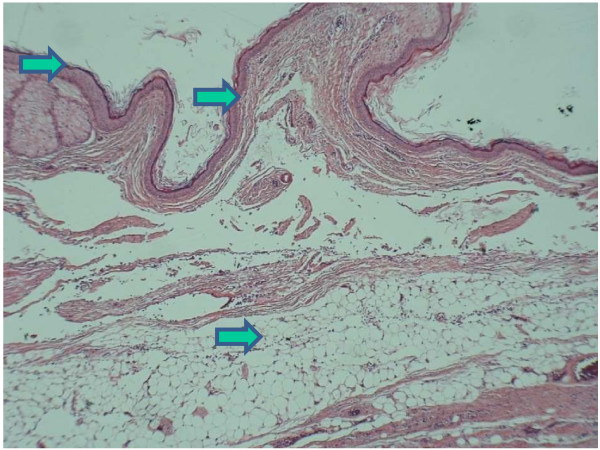
**Histological slide showing lining of the cyst**, **skin and adipose tissue confirming the diagnosis of a dermoid cyst.**

A booking ultrasound scan at 13 weeks gestation in a 27-year-old primigravida showed an 11 × 9 × 10 cm smooth-walled, mixed echogenic mass in the pouch of Douglas (POD) suggesting a dermoid cyst and a viable singleton intra-uterine pregnancy. A repeat scan four weeks later showed an increase in the size of the cyst of 1 cm in each dimension. CA-125 assay was within the normal limits in pregnancy. Alpha-foetoprotein (AFP) was not tested owing to the difficulty in interpretation during pregnancy. Following extensive discussion of different treatment options and timings, the patient opted for surgical removal. The left ovary together with the cyst was removed at 18 weeks of pregnancy. The cyst was impacted in the pelvis owing to the gravid uterus and unfortunately it ruptured during removal. The peritoneal cavity was washed meticulously with 0.9% saline solution. In view of the almost definite clinical diagnosis of an ovarian dermoid cyst, the peritoneal fluid was not sent for cytology. The right ovary was examined per-operatively and looked clinically normal. Histology of the cyst confirmed the diagnosis of a mature cystic teratoma (figure 1). The patient was discharged on the sixth postoperative day. She presented again at 20 weeks gestation with abdominal pain, breathlessness and generalised malaise. Investigations were performed including a VQ (ventilation and perfusion) scan that was highly suggestive of pulmonary embolism. She was treated with low-molecular-weight heparin. She continued to have episodes of abdominal pain and fluctuating temperature. The white cell count range was 9–19 × 10^9^/l and the C-reactive protein range was 76–163.4 ng/dL. Repeated bacteriological cultures were negative.

A magnetic resonance imaging (MRI) scan showed a collection of fluid occupying the POD and subsequently a subcutaneous collection was found during an ultrasound scan. She was treated with antibiotics empirically. Seven weeks following her initial surgery, she developed painful induration of the wound. She underwent wound exploration that revealed copious, thick, sebaceous-type material tracking extra-peritoneally up to the costal margin and umbilicus. Bacterial culture was again reported to be negative.

She developed dependent oedema, abdominal distension, intermittent vomiting and significant weight loss despite advancing pregnancy. In view of the patient's continuing deterioration and significant psychological distress, it was decided to deliver the baby.

**Figure 2:**
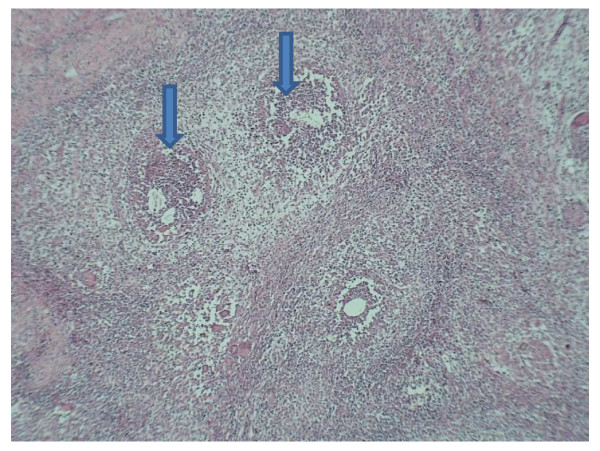
Histological slide showing chronic granulomatous reaction.

She underwent an elective caesarean section at 31 weeks gestation following a course of antenatal steroids for foetal pulmonary maturation. A caesarean section was performed under epidural anaesthesia in the presence of a general surgeon. The anterior abdominal wall was morbidly adherent to the uterus making identification of the uterus virtually impossible. Eventually the 'lower segment' was exposed with the fundus remaining obscured by the dense adhesions. The caesarean section was performed with a J-shaped incision. The baby was delivered in good condition. The patient underwent general anaesthesia following delivery. There were diffuse adhesions with the bowel adhered to the uterine fundus and lower part of the pelvis. There were large lumps of possible granulation tissue on the bowel clinically mimicking malignancy. The bowel was compromised with adhesions producing kinking and bowel stasis. About 85 cm of the ileum and 45 cm of the caecum and colon involved with the mass suggesting infiltrating malignancy were resected by colorectal surgeons. The abdominal wall, rectus muscles, sheath and bowel were infiltrated by sebum-like material. Following extensive exploration and drainage, the patient was left with a mucus fistula on the left side of the abdomen and an ileostomy on the right side. Histology of the specimen confirmed granulation and inflammation consistent with granulomatous peritonitis without any evidence of malignancy (figure 2). She suffered further wound breakdown and developed a small bowel fistula through the operative wound. She also developed anaemia, hypoproteinaemia, bilateral pedal oedema and intermittent episodes of abdominal pain with dyspnoea. A computed tomography (CT) scan showed a homogenous collection in the POD. An attempt to drain this possible collection vaginally was not successful. In view of her continuing pain and poor nutritional status, advice was sought from a tertiary referral centre. Discussions and journal searches suggested that this rare condition settles spontaneously and steroids might be of some help. In an experimental model with rats, a massive granulomatous reaction induced by intra-peritoneal inoculation of starch was significantly reduced when prednisone at a dosage of approximately 1 mg/kg orally daily was commenced two weeks before inoculation. Therapy started when the starch was inoculated had only a minor effect on this response, but if commenced two weeks after inoculation, it failed to ameliorate the granulomatous reaction [6].

Over the following six weeks (one year after her initial surgery), her condition improved both physically and mentally. The inflammatory markers returned to normal. She is presently thinking about going back to work and the possibility of conceiving again.

## Discussion

The word teratoma is derived from the Greek word *teraton*, meaning monster, and was used initially by Virchow in the first edition of his book on tumours, which was published in 1863 (see [2]). Since mature cystic teratomas are composed of all three germ cell layers, the term 'dermoid' is a misnomer. Mature cystic teratomas are thought to arise from primordial germ cells. This theory is supported by the distribution of these tumours along the lines of migration from the yolk sac to the primitive gonad. The majority of these tumours occur during the reproductive years providing further support for the germ cell theory [2]. Benign cystic teratoma (BCT) is the most common benign ovarian neoplasm comprising 10–15% of all ovarian tumours. It occurs at all stages of life, the majority of cases being diagnosed between 20 and 30 years of age [4]. This fact makes it the most common tumour during pregnancy (22–40% of all ovarian tumours) [4]. In pregnancy the risk of complications increases significantly including rupture, torsion, infection and malignant degeneration. As BCT has a tendency to remain in the confines of the true pelvis, it could lead to dystocia and obstructed labour [4]. Treatment is surgical removal as soon as possible after diagnosis to avoid complications. Ovarian cystectomies or oophorectomies via laparoscopy or laparotomy are options depending on the situation and expertise available. All efforts should be made to avoid rupture or leakage of cyst fluid during the operation. If it happens before or during the operation, copious saline washing should be performed to minimise chemical peritonitis and its sequelae [3,7,8]. When BCT is found incidentally in the first trimester of pregnancy, surgical removal should be performed at 14–16 weeks of gestation to avoid the risk of damage to the corpus luteum. If diagnosis of BCT occurs at 16–22 weeks, surgery should be performed as soon as possible. If it is first discovered after 22 weeks of pregnancy, the treatment may be deferred until delivery [4]. Over 200 cases of BCT in pregnancy have been reported in the literature and many of them ruptured spontaneously or iatrogenically. In a review of 47 cases, Kocak et al [9] reported that during cyst extraction, minimal spillage occurred in 42.5% of cases and none developed chemical peritonitis.

Clement et al [7] and Achtari et al [8] reported chemical peritonitis following cystic fluid spillage. The patients needed further surgeries to treat the complications. Two other case reports by Suprasert et al [4] and one by Phupong et al [5] reported a diffuse peritoneal reaction mimicking advanced ovarian malignancy where full surgical staging was performed. Postoperative histological examination revealed BCT and chronic granulomatous peritonitis. A conservative approach was adopted and patients were free of symptoms without further treatment at 12 months [1,5]. The incidence of chemical peritonitis after rupture and leakage of cystic fluid in the peritoneum is less than 0.5% (see [2]). The incidence of chronic granulomatous peritonitis after rupture or leakage of cystic fluid is also extremely rare. In granulomatous peritonitis after ruptured ovarian teratomas, numerous nodules of mature glial tissue implant on a widespread area of the peritoneum [10].

Ruptured BCT of the ovary mimicking gynaecological malignancy is uncommon and could be misdiagnosed [5]. Intra-abdominal peritoneal seedlings, adhesions and/or masses are frequent sequelae. In most such cases, abdominal seedlings are essentially of mature neuroglial elements and long-term survival rate is good. Recognition of a dermoid tumour associated with glial seedling is important to avoid unnecessary debulking surgery. Following postoperative adhesions, fibrous bands or obstructions, conservative management seems to have a good prognosis.

## Conclusion

Although granulomatous peritonitis following rupture of a dermoid cyst is very rare, awareness is the key to diagnosis and appropriate management. Peroperative frozen section may be helpful. Optimal management avoids unnecessary repeated major surgeries and provides a good long-term outcome with minimal complications.

## Competing interests

The authors declare that they have no competing interests.

## Consent

Written informed consent was obtained from the patient for publication of this case report. A copy of the written consent is available for review by the Editor-in-Chief of this journal.

## Authors' contributions

SM and ZF collected the data, and designed the case report with significant contributions from ZKA and REH, SM formatted and finalised the draft. All authors read and approved the final manuscript.

## References

[B1] Comerci JT Jr Licciardi F Bergh PA Gregori C Breen JL Mature cystic teratoma: a clinicopathologic evaluation of 517 cases and review of the literature Obstet Gynecol 1994 84 22 28 8008317

[B2] Peterson WF Prevost EC Edmunds FT Hundley JM Jr Morris FK Benign cystic teratomas of the ovary; a clinico-statistical study of 1,007 cases with a review of the literature Am J Obstet Gynecol 1955 70 368 382 13238472 10.1016/s0002-9378(16)37681-5

[B3] Stuart GC Smith JP Ruptured benign cystic teratomas mimicking gynecologic malignancy Gynecol Oncol 1983 16 139 143 10.1016/0090-8258(83)90019-7 6884826

[B4] Suprasert P Khunamornpong S Siriaunkgul S Phongnarisorn C Siriaree S Ruptured mature cystic teratomas mimicking advanced stage ovarian cancer: a report of 2 cases study J Med Assoc Thai 2004 87 12 20041522 1525 15822552

[B5] Phupong V Sueblinvong T Triratanachat S Ovarian teratoma with diffused peritoneal reactions mimicking advanced ovarian malignancy Arch Gynecol Obstet 2004 270 189 191 10.1007/s00404-003-0479-8 15526210

[B6] Cade D Ellis H The peritoneal reaction to starch and its modification by prednisone Eur Surg Res 1976 8 471 479 10.1159/000127891 991891

[B7] Clement D Barranger E Benchimol Y Uzan S Chemical peritonitis: a rare complication of an iatrogenic ovarian dermoid cyst rupture Surg Endosc 2003 17 658 10.1007/s00464-002-4272-8 12574929

[B8] Achtari C Genolet PM Bouzourene H De Grandi P Chemical peritonitis after iatrogenic rupture of a dermoid cyst of the ovary treated by coelioscopy. Apropos of a case and review of the literature Gynakol Geburtshilfliche Rundsch 1998 38 3 146 150 10.1159/000022253 9885355

[B9] Kocak M Dilbaz B Ozturk N Dede S Altay M Dilbaz S Haberal A Laparoscopic management of ovarian dermoid cysts: a review of 47 cases Ann Saudi Med 2004 24 357 360 15573848 10.5144/0256-4947.2004.357 PMC6148138

[B10] Ronnett BM Seidman JD Mucinous tumors arising in ovarian mature cystic teratomas: relationship to the clinical syndrome of pseudomyxomaperitonei Am J Surg Pathol 2003 27 650 657 10.1097/00000478-200305000-00008 12717249

